# Supplementary material for: The genome sequence of Lathbury’s Nomad Bee, Nomada lathburiana (Kirby, 1802)
Source: Wellcome Open Res. 2025 Apr 23;10:211. [Version 1] doi: 10.12688/wellcomeopenres.24013.1 (PMC12177452; doi:10.12688/wellcomeopenres.24013.1)
Supplement: Peer review history

# Reviewer report: Reviewer response for version 1

**DOI:** 10.21956/wellcomeopenres.26493.r125654

**Published:** 2025-06-18

**Authors:** Jason Charamis

**Affiliations:** 1 Foundation for Research and Technology - Hellas, Irákleion, Greece

This paper presents the genome assembly of Lathbury's Nomad Bee ( *Nomada lathburiana*), a parasitic bee species from the family Apidae. The work is technically sound. The paper follows established protocols from the Darwin Tree of Life consortium, ensuring reproducibility and standardization. Quality assessment using multiple tools (BUSCO, Merqury, k-mer analysis) provides comprehensive validation. The use of Hi-C data for chromosome-level scaffolding represents current best practices in genome assembly. Sample collection and DNA barcoding procedures appear robust, with proper species verification through COI sequencing.

 My only suggestion is to add more background on this organism, why we study it and what relevance could it have in terms of ecological studies.

Are sufficient details of methods and materials provided to allow replication by others?

Yes

Is the rationale for creating the dataset(s) clearly described?

Yes

Are the datasets clearly presented in a useable and accessible format?

Yes

Are the protocols appropriate and is the work technically sound?

Yes

Reviewer Expertise:

Arthropod Comparative Genomics

I confirm that I have read this submission and believe that I have an appropriate level of expertise to confirm that it is of an acceptable scientific standard.

# Reviewer report: Reviewer response for version 1

**DOI:** 10.21956/wellcomeopenres.26493.r125041

**Published:** 2025-06-18

**Authors:** Christoph Bleidorn

**Affiliations:** 1 University of Göttingen, Göttingen, Germany

*Nomada lathburiana* is a cleptoparasitic bee that lays its eggs in the nests of the mining bee *Andrena cineraria*, relying on the host’s pollen stores to feed its larvae. It is typically active in spring and found in sunny, flower-rich habitats such as grasslands and woodland edges, where its host species also nests. The speciment was ID`d by an expert in the field. The genome was sequenced and assembled for the DToL project, which its typical high standard of methods. Most of the assembly could be assigned to 15 scaffolds representing different chromosomes. As for other nomad bees sequenced with long-read techniques, an unusually large mitochondrial genome was reported. Some more information or statistics on its properties would be welcome. Otherwise, I fully recommend publication of this report.

Are sufficient details of methods and materials provided to allow replication by others?

Yes

Is the rationale for creating the dataset(s) clearly described?

Yes

Are the datasets clearly presented in a useable and accessible format?

Yes

Are the protocols appropriate and is the work technically sound?

Yes

Reviewer Expertise:

phylogenomics of annelids, Hymenoptera and bacterial symbionts

I confirm that I have read this submission and believe that I have an appropriate level of expertise to confirm that it is of an acceptable scientific standard.
